# Characterizing Socioecological Markers of Differentiated HIV Risk Among Men Who Have Sex with Men in Indonesia

**DOI:** 10.1007/s10461-023-04253-3

**Published:** 2024-01-25

**Authors:** Laura Nevendorff, Alisa Pedrana, Adam Bourne, Michael Traeger, Eric Sindunata, Wawa A. Reswana, Rosidin M. Alharbi, Mark Stoové

**Affiliations:** 1 Disease Elimination Program, Burnet Institute, 85 Commercial Rd, Melbourne, 3004 Australia; 2 School of Public Health and Preventive Medicine, Monash University, Melbourne, Australia; 3 HIV AIDS Research Center Atma Jaya Catholic University Jakarta, Jakarta, Indonesia; 4 Australian Research Centre in Sex, Health and Society, La Trobe University, Melbourne, Australia; 5 Kirby Institute, University of New South Wales, Sydney, Australia; 6 Jaringan Indonesia Positive (The Positive Indonesia Network), Jakarta, Indonesia

**Keywords:** HIV risks, Socioecological risks, Co-occurring risks, Men who have sex with men, Indonesia, Latent class analysis

## Abstract

HIV prevention programs typically focus on changing individuals’ risk behaviors, often without considering the socioecological factors that can moderate this risk. We characterized HIV risk among men who have sex with men (MSM) in Indonesia (n = 1314) using latent class analysis and used multinomial logistic regression to identify latent class relationships with demographics, social/sexual networks, and community-level socioecological indicators of HIV risk. Three HIV risk latent classes were identified—“Sexually Moderate” (n = 333), “Sexual Explorative” (n = 575), and “Navigating Complexities” (n = 406). Using “Sexually Moderate” (lowest risk) as the reference group, MSM in the “Sexual Explorative” class had additional social/sexual network-level risks (meeting partner(s) using both online and offline methods [RR = 3.8; 95%CI 1.7–8.6] or general social media and gay-specific online platforms [RR = 2.6; 95%CI 1.9–3.6] to meet partners, group sex [RR = 10.9; 95%CI 4.5–25.4], transactional sex [RR = 1.6; 95%CI 1.2–2.2]), and community-level risks (experiencing homosexual-related assaults [RR = 1.4; 95%CI 1.1–1.9]). MSM in the “Navigating Complexities” class had additional social/sexual network-level risks (low social support [RR = 1.6; 95%CI 1.1–2.5], less disclosure of their sexuality [RR = 1.4; 95%CI 1.0–1.9]) and community-level risks (higher internalized homonegativity scores [RR = 1.2; 95%CI 1.1–1.4], ever experiencing homosexual-related assaults [RR = 1.4:95%CI 1.1–1.9], less exposure to HIV/STI health promotion [RR = 0.7; 95%CI 0.5–0.9], attending STI-related services in the past 6 months [RR = 0.6; 95%CI 0.4–0.8]). Co-occurring individual and socioecological risk recommend holistic HIV prevention strategies tailored to consider the social and structural conditions of MSM in Indonesia are needed.

**Supplementary Information:**

The online version contains supplementary material available at 10.1007/s10461-023-04253-3.

## Introduction

Men who have sex with men (MSM) account for approximately 45% of global HIV incidence outside of Africa, with an estimated 26-fold higher risk of HIV infection than non-MSM [1]. In the absence of effective prevention strategies, sexual practices are the primary route of HIV transmission among MSM [2]. Consequently, most HIV prevention programs focus on risk education and individual-level behavior change to promote the use of condoms, HIV pre-exposure prophylaxis (PrEP) and treatment as prevention [3–5].

However, HIV risk is also shaped by contextual social and structural factors outside of an individual’s control [6]. The modified social-ecological model of HIV risk provides a useful framework for investigating multi-level risks and risk contexts beyond those associated with individual-level behaviors [7]. The framework explains the interplay between individual-level risks (i.e., risk behaviors), social and sexual network-level risks (i.e., interpersonal relationships) and community-level risks (i.e., environments in which risk behaviors may be more or less likely to occur). For MSM, social-ecological HIV risk manifests in many ways, including via sexual networks [8] and the influence of geospatial, physical [9] and online spaces that form sexual networks and may potentiate risk [10]. Furthermore, stigmatizing social, cultural and political norms and the creation of minority stress limits engagement with preventive and other HIV services to facilitate risk. These social and structural factors combine with individual factors to shape sexual risk behaviors among MSM.

In Indonesia, where prevalence of HIV among MSM is estimated to be as high as 18% [11], a complex interplay between community, structural and societal factors influence individual-level sexual risk behaviors among MSM. Extrinsic influences are particularly shaped by increasing Islamic conservatism, which is becoming more visible in Indonesian politics, courts and local policies [12] and resulting in stricter regulations related to gender and sexuality norms [13]. As a result, gay venue raids and mass media condemnation of homosexuality have become more common [14]. In addition, the wide use of online social networks to meet sexual partners facilitates sexual risk behavior in this group [15]. This creates an environment that limits disclosure of risk practices (e.g., multiple sex partners [16], sexualized drug use (SDU) [17]), and missed opportunities to access HIV prevention and other sexual health programs for MSM [18]. This becomes more problematic in the context of low levels of HIV viral suppression, inadequate knowledge of HIV prevention and treatment, and poor coverage of HIV PrEP [19].

Despite the socioecological and HIV-related epidemiological and behavioral contexts for MSM in Indonesia [20], there has been little research on their combined contribution to shaping overall HIV risk. Most studies of MSM in Indonesia have focused on describing individual behaviors as drivers of risk, such as condomless anal intercourse (CAI) as a driver of HIV risk, the role of HIV status in influencing risk behaviors, and the effect of HIV knowledge on unsafe sex [16, 21, 22]. Some studies have begun to recognize the contribution of social and structural factors to HIV risk [23], but have not directly assessed their links with individual risk behaviors and sexual practices.

While specific individual-level behaviors, such as CAI, are typically well defined in terms of biological plausibility of HIV transmission, their influence on population-level HIV prevalence and incidence (and therefore their priority for HIV prevention policy and practice) is often dependent on intersecting and context-specific factors. Focusing on a specific HIV risk behavior is therefore fraught with the possibility of ignoring factors that may otherwise be crucial for effective HIV prevention policy and practice. Latent class analysis (LCA) is a statistical method that can consider multiple risk factors simultaneously, distilling them down to emergent and unobserved risk constructs. As such, LCA provides a way to identify a range of underlying HIV risk characteristics that can, in turn, be used to examine how more nuanced definitions of risk are differentiated by social and structural factors [24, 25]. To help inform HIV prevention priorities and tailored programs for MSM in Indonesia, we used cross-sectional survey data collected from the Chemsex-INA study to (i) profile the patterns of HIV-related sexual risk practices among MSM in Indonesia using LCA; and (ii) assess whether the patterns of HIV risk characterized in the latent classes were associated with individual demographics, social and sexual networks, and community-level socioecological risk factors using multinomial logistic regression models.

## Methods

### Study Population and Procedure

This study used data from the Chemsex-INA study—a community-led national online survey initiated by the national network of gay men, transgender people, and other MSM who do not openly identify as homosexual in Indonesia (*Gaya Warna Lentera Indonesia*—GWL-INA), in collaboration with the HIV/AIDS research center Atma Jaya Catholic University Jakarta, Indonesia (ARC). Study participants completed self-administered online surveys between July 29th and October 9th, 2019. The inclusion criteria for participating in the study were self-reporting male gender and reporting ever having sex and/or sexual arousal with men, being at least 18 years old and living in Indonesia. The Chemsex-INA questionnaire was adapted from the European MSM internet survey (EMIS-2017), [26] with questions targeting demographics, health, risks and precautions for HIV or sexually transmissible infections (STIs), and service access domains. Some adjustments to questions associated with drug use were made based on prior qualitative studies that documented distinct local patterns of drugs utilized, SDU frequencies, SDU motivations and source of drug(s) for SDU practice. Using a network sampling approach, the survey link banner was promoted through online strategies (GWL-INA’s members and their social media, website and social media of GWL-INA and ARC and selected social media influencers) and promoted by key MSM community members and outreach workers in several Indonesian cities to their networks. Participants received no direct compensation for their participation, but those who completed the survey were eligible to win one of 15 “door prize gifts” worth between USD6.7 and USD10. The study was approved by the Ethics Committee of Atma Jaya Catholic University Jakarta Indonesia (No. 0401/III/LPPM-PM.10.05/04/2019), with additional ethics approvals from the Alfred Ethics Committee in Melbourne (No. 421/20) for data sharing, storage and analysis in Australia. This article follows the STROBE guidelines for reporting of observational cross-sectional studies [27].

In this analysis, data were restricted to self-identified cisgender MSM who had at least one male sex partner in their lifetime who completed the Chemsex-INA questionnaire. Transgender women were excluded due to sociodemographic and HIV-related sexual and structural risk determinants that differ from those of cisgender MSM [11, 28].

### Measurement

The selection of variables used to define both individual risk in the LCA and associated socio-ecological risk was based on the modified social ecological model proposed by Baral et al. [7]. This model emphasizes the connection between proximal individual-level risks and higher-order social and structural-level risks when considering population-level HIV prevention approaches, and is therefore well suited to the socio-ecological context of HIV in Indonesia.

#### Individual HIV Risk Indicators

*Individual HIV risk factors* were defined as biological or behavioral characteristics linked to susceptibility to acquire or transmit HIV [7]. To determine individual-level patterns of HIV risk, in the LCA we assessed the presence or absence of indicators of HIV risk identified in previous studies of HIV among MSM in Indonesia [22, 23]. The seven indicators were: self-reported HIV positive status (no/yes); anxiety and depression (moderate or severe classified according to the ultra-brief screening scale PHQ-4 tool [29] (no/yes); number of male sex partners in the past month (no sexual intercourse/one/2+); consistent condom use with male regular partner(s) in the past 12 months (yes/no/no regular partner); consistent condom use with male casual partner(s) in the past month (yes/no/no casual partner(s)); lifetime drug use during or before sex (never/single drug/polydrug; excludes alcohol and erectile dysfunction drugs); and ever been diagnosed with any STI (never/ever/never been tested).

#### Socioecological Factors

We selected key variables from the survey that characterized individual-level demographics (including level of HIV knowledge), and social and sexual network-level and community-level risk factors based on operational knowledge of the socio-political and structural context for HIV in Indonesia (the first author is an experienced HIV prevention program specialist in Indonesia) to construct a socioecological HIV risk model [7].

*Individual-level demographic* factors were defined as a set of basic conditions for MSM that may directly or indirectly affect their likelihood to engage in specific sexual risk. Six individual-level demographic variables included in the analysis were: age groups (≤ 24/≥ 25 years old), education levels (≤ high school/≥ college); monthly income (approximately < USD200/USD200–669/> USD669); area of residency (districts/city); employment status (unemployed/employed, student); and level of HIV knowledge (low/medium/high). HIV knowledge was determined from six 5-response questions (yes/no/I wasn’t sure about this/I don’t understand/I do not believe this) about HIV prevention, transmission, and treatment, which were categorized as either correct (correct yes/no response) or incorrect (incorrect yes/no response or any other response). Participants were categorized as having low, medium, or high knowledge if they answered 1–3 questions, 4–5 questions, or all questions correctly, respectively.

*Social and sexual network* risk factors were defined as factors that influence the micro-environments in which MSM interact, share information, provide and/or receive social support, and meet sex partners, and thus may modify risk of HIV acquisition [30]. Six social and sexual network-level risk factor variables included in the analysis were: method of meeting sex partner(s) (offline only/online only/combination of offline and online/preferred not to answer); online social network platform utilization (general social media only/combination of general social media and gay-specific dating apps); low social support (no/yes, with low social support classified by scores below 10 on either ‘Reliable Alliance’ or ‘Social integration’ subscales of the Social Provision Scale) [31]; engaged in group sex during last sex act (no/yes); open disclosure of sexuality (no/yes); and ever engaged in selling or buying sex (no/yes).

*Community-level* risk factors were defined as factors that influence the relationships MSM have with community and HIV services that have the potential to mediate HIV risk [7]. Five community-level risk factor variables included in the analysis were: internalized homonegativity (scored from 0 to 6 on the Internalized Homonegativity Scale) [32]; ever experienced homosexual-related assaults (no/yes); received HIV/STI-related health promotion information for MSM in the past 6 months (no/yes); attended an HIV-related service in the past 6 months (no/yes); and attended STI-related services in the past 6 months (no/yes).

### Statistical Analysis

Descriptive statistics were generated for participants’ socio-demographics. The study performed a two-step analysis. First, LCA was used to explore unobserved heterogeneity in HIV risk. Classes of HIV risk were created based on the clustering of the individual HIV risk indicators described above [33]. We used maximum likelihood estimation in generalized structural equation modelling to randomly divide selected indicators into classes and reclassified until the best model fit was found. Starting values were computed using random classes assignments. We specified 100 random guesses for class probabilities, with 30 expectation–maximization iterations for each random draw to identify the best model fit for 2–5 classes. The minimum allowed class size was restricted to 10% of the sample. The final number of classes was determined on the basis of lowest Akaike Information Criterion (AIC) and Bayesian Information Criterion (BIC) values, greatest model entropy, and manual inspection to ensure interpretability. We compared models containing 2–5 latent classes to determine the optimal fit. On the basis of AIC and BIC fit, as well as entropy criteria (see Supporting Information Table 1) and interpretability considerations, we determined a three-class model (AIC = 12,855, BIC = 13,036, Entropy = 0.84) best delineated the data.

We predicted the posterior probability of belonging to the specific subgroup classes and classified participants accordingly. Conditional independence was assessed by computing Pearson’s correlation coefficient for all variables within each class, with none of the variables indicating a violation of the independence assumption (correlation of greater than 0.5).

We used descriptive statistics and Pearson chi-squared test for categorical outcomes and one-way ANOVA for continuous outcomes to characterize differences across socioecological factors between individuals belonging to each class. Comparative analysis of socioecological factors using multinomial logistic regression was performed to identify factors associated with class membership, with the class determined to exhibit lowest sexual risk as a reference group. We calculated the adjusted relative risk ratio (RR) and the respective 95% confidence interval (CI). Stata version 17 (Stata Corp, College Station, TX, USA) was used to analyze all data.

## Results

### Demographic Characteristics

In total, 1412 individuals completed the Chemsex-INA survey, of whom eight (0.6%) identified as female, 23 (1.6%) as transgender female, and 67 (4.7%) as never having had sex with men; these 98 individuals were excluded from this analysis. Among 1314 MSM included, the median age was 28 years (interquartile range [IQR] 23–34), just over half reported completing diploma education level or above, just less than half reported a monthly income below USD207, nearly two thirds lived in a metropolitan area and more than four fifths were employed at the time of survey completion (Table 1).

**Table 1:** Sociodemographic characteristics of participants

Characteristics	Value (N = 1314)	
Age, year (median, IQR)	28	25–34

	Freq	%
Age group		
24 years old or below	319	24.3
25 years old or above	995	75.7
Education level		
High school or below	564	42.9
College or above	750	57.1
Monthly income		
< USD207	635	48.3
USD207–669	531	40.4
> USD669	148	11.3
Residential		
District area	467	35.5
Metropolitan area	847	64.5
Employment status		
Unemployed	98	7.5
Employed*	1117	85.0
Student	99	7.5

*Including: full-time, part-time, and self-employed

### Latent Class Model: Patterns of Individual HIV Risks

The three distinct classes of MSM based on individual HIV risk are presented in Table 2. MSM in the first class (n = 333, 26.2%) were generally at lowest risk of HIV acquisition and were more likely to report sex with one person in the past month, sex with regular sex partners, and no sex with casual partners in the past 12 months, while being less likely to report lifetime SDU; hereafter referred to as the *Sexually Moderate* group. MSM in the second class (n = 575, 43.9%) were more likely to report more than one sex partner and inconsistent condom use with casual partners in the past month, report lifetime SDU using single or multiple drugs, and report ever being diagnosed with an STI; this group is hereafter referred to as the *Sexual Explorative* group. MSM in the third class (n = 406, 29.8%) were more likely to report being HIV positive, being categorized as having moderate to severe anxiety and depression, report no sex with regular or casual partners in the past month and having never had an STI test; this group is hereafter referred to as the *Navigating Complexities* group.

**Table 2:** Distribution of HIV risks factors by latent class membership (N = 1314)

	Overall	*Sexually moderate* Group (class 1)	*Sexual explorative* Group (class 2)	*Navigating complexities* Group (class 3)
	N (%)	n = 333	n = 575	n = 406
Unconditional probability of each class		0.262	0.439	0.298
Self-reported HIV positive status	561 (42.7)	0.369	0.410	0.501
Had moderate/severe anxiety and depression	222 (16.89)	0.135	0.160	0.210
Number of male sex partners in the past month				
No sexual intercourse	425 (32.3)	0	0.067	0.984
1 person	506 (38.5)	0.936	0.313	0.006
2+ people	383 (29.2)	0.063	0.619	0.009
Consistent condom uses with male *regular* partner(s) in the past 12 months				
Yes	413 (31.43)	0.377	0.288	0.297
No	572 (43.53)	0.565	0.443	0.308
No regular partner(s)	329 (25.04)	0.056	0.268	0.394
Consistent condom use with male *casual* partner(s) in the past month				
Yes	76 (5.78)	0.056	0.077	0.029
No	601 (45.74)	0.080	0.878	0.169
No casual partner(s)	637 (48.48)	0.863	0.044	0.800
Pattern of lifetime drug use during or before sex				
Never	979 (74.5)	0.818	0.635	0.843
Single drug	215 (16.4)	0.114	0.228	0.110
Polydrug	120 (9.1)	0.067	0.135	0.047
Ever been diagnosed with any STI				
Never	471 (35.8)	0.420	0.327	0.348
Ever	497 (37.8)	0.376	0.447	0.278
Never get tested	346 (26.3)	0.202	0.225	0.373

### Socioecological Risks by Classes of Sexual Risk Behavior

Table 3 describes demographic and socio-ecological risk characteristics of MSM within each of the three classes. MSM across the three classes had mostly similar sociodemographic characteristics and HIV knowledge, but MSM in the *Navigating Complexities* group reported lower monthly income and were less likely to reside in a metropolitan area. MSM in the *Sexually Moderate* class were more likely to meet sexual partners offline only, utilize general social media only, receive more social support, and be exposed to HIV/STI health promotion in the past 6 months. They were less likely to engage in group sex, experience homosexual-related assaults, and attend STI-related services in the past 6 months. MSM in the *Sexual Explorative* group were more likely to utilize both online and offline methods to meet sexual partner(s) and use both social media and gay-specific dating apps, and report group sex during their last sex act, selling or buying sex, and being more open about their sexuality. MSM in the *Navigating Complexities* group were more likely to report low social support, were less open about their sexuality, scored higher for internalized homonegativity, and less likely to receive HIV/STI health promotion and attend STI-related services in the past 6 months.

**Table 3:** Distribution of demographic characteristics, HIV-related individual, social and community factors by latent class membership and tests for differences between subgroups

Variables	*Sexually moderate* Group (class 1)	*Sexual explorative* Group (class 2)	*Navigating complexities* Group (class 3)	Test for difference^a^
	%	%	%	
Domain 1: individual-level demographic				
Age group				0.461
24 years and below	25.5	22.6	25.6	
25 years and above	74.5	77.4	74.4	
Education level				0.056
High school or below	46.8	39.3	44.8	
Diploma certificate or above	53.2	60.7	55.2	
Monthly income				** < 0.001**
< USD 207	46.8	43.0	57.1	
USD 207–669	42.0	42.4	36.2	
> USD 669	11.1	14.6	6.7	
Residential area				** < 0.001**
District	35.1	30.1	43.6	
City	64.9	69.9	56.4	
Employment status				0.083
Unemployed	6.3	6.1	10.3	
Employed*	85.6	87.1	81.5	
Student	8.1	6.8	8.2	
Level of HIV knowledge				0.931
Low	18.0	18.4	20.0	
Medium	56.5	56.0	56.4	
High	25.5	25.6	23.6	
Domain 2: social and sexual network factors				
Method of meeting sex partner(s)				** < 0.001**
Offline only	8.7	2.1	6.9	
Online only	67.0	77.7	74.4	
Combination of online & offline	12.3	19.1	12.1	
Preferred not to answer	12	1.1	6.6	
Online platform utilization				** < 0.001**
General social media only	58.6	27.5	57.4	
Social media & gay-specific dating apps	41.4	72.5	42.6	
Low social support				**0.026**
No	89.2	84.0	82.3	
Yes	10.8	16.0	17.7	
Engaged in group sex during last sex act				** < 0.001**
No	98.2	78.3	90.1	
Yes	1.8	21.7	9.9	
Fully or moderate disclosure of sexuality				** < 0.001**
No	60.7	51.8	69.5	
Yes	39.4	48.2	30.5	
Ever engaged in selling or buying sex				** < 0.001**
No	66.1	48.3	68.5	
Yes	33.9	51.7	31.5	
Domain 3: community-level factors				
Internalized homonegativity index (continuous var.)	2.81	2.8	3.25	** < 0.001**
Ever experienced homosexual-related assaults				**0.051**
No	60.4	52.0	54.7	
Yes	39.6	48.0	45.3	
Received HIV/STI health promotion information for MSM in the past 6 months				** < 0.001**
No	23.4	25.0	35.2	
Yes	76.6	75.0	64.8	
Attended HIV-related services in the past 6 months				**0.008**
No	24.3	24.3	32.5	
Yes	75.7	75.7	67.5	
Attended STI-related services in the past 6 months				** < 0.001**
No	46.3	47.7	53.3	
Yes	53.7	53.3	36.7	

Bold values indicate statistically significant at P < 0.05

*STI* sexual transmission infections

*Including: full-time, part-time, and self-employed

^a^Pearson Chi2 test were for categorical variables and one-way ANOVA for continuous variable

Using *Sexually Moderate* as the reference group, we assessed the relative risk of the *Sexual Explorative* and *Navigating Complexities* groups within each of the socioecological domains. Those in the *Sexual Explorative* group had significantly higher risk in the social and sexual networks domain, including methods to meet sexual partner(s) (online only RR = 4.3; 95%CI 2.1–8.9, and both online and offline methods RR = 3.8; 95%CI 1.7–8.6), use of both general social media and gay-specific dating platforms (RR = 2.6; 95%CI 1.9–3.6), and ever selling or buying sex (Table 4) (RR = 1.6; 95%CI 1.2–2.2). In contrast, those assigned to the *Navigating Complexities* group were less likely to report higher monthly income (> 699 USD RR = 0.5; 95%CI 0.3–0.9) and, in the sexual network domain, more likely to report low social support (RR = 1.6; 95%CI 1.1–2.5) and less disclosure of their sexuality (RR = 1.4; 95%CI 1.0–1.9). Within the community-level domain, the *Navigating Complexities* group scored higher on internalized homonegativity, index (RR = 1.2; 95%CI 1.1–1.4) and had less access to HIV/STI health promotion information (RR = 0.7; 95%CI 0.5–0.9) and were less likely to have attended STI-related services in the past 6 months (RR = 0.6; 95%CI 0.4–0.8). Relative to the *Sexually Moderate* group, the *Sexual Explorative* and the *Navigating Complexities* groups had higher risk associated with engaging in group sex (RR = 10.9; 95%CI 4.5–25.4 and RR = 6.1; 95%CI 2.5–14.5, respectively) and ever experiencing homosexual-related assaults (RR = 1.4; 95%CI 1.1–1.9 and RR = 1.3; 95%CI 1.1–1.9, respectively) (Table 4).

**Table 4:** Multinomial logistic model relating respondents’ socioecological risk factors to HIV risk patterns (class 1 as ref)

	Class 2			Class 3		
	*Sexual explorative*			*Navigating complexities*		
	Group			Group		
	RR	(95%CI)	P value	RR	(95%CI)	P value
Domain 1: individual-level demographic factors						
Age group						
24 years and below	Ref			Ref		
25 years and above	1.1	(0.7–1.5)	0.747	1.1	(0.8–1.7)	0.522
Education level						
High school or below	Ref			Ref		
Diploma certificate or above	1.3	(0.9–1.7)	0.112	1.3	(0.9–1.8)	0.103
Monthly income						
< USD207	Ref			Ref		
USD207–669	0.9	(0.7–1.3)	0.912	0.7	(0.5–1.0)	0.056
> USD669	1.2	(0.7–1.9)	0.518	**0.5**	**(0.3–0.8)**	**0.009**
Residential area						
District	Ref			Ref		
City	1.2	(0.9–1.6)	0.213	0.7	(0.5–1.0)	0.079
Employment status						
Unemployed	Ref			Ref		
Employed*	0.9	(0.5–1.8)	0.962	0.7	(0.4–1.3)	0.232
Student	0.9	(0.4–1.9)	0.858	0.7	(0.3–1.5)	0.365
Level of HIV knowledge						
Low	Ref			Ref		
Medium	0.9	(0.6–1.3)	0.664	0.9	(0.6–1.4)	0.721
High	0.9	(0.6–1.4)	0.749	0.8	(0.5–1.4)	0.593
Domain 2: social and sexual network factors						
Ways of meeting sex partner						
Offline only	Ref					
Online only	**4.3**	**(2.1–8.9)**	**0.001**	1.3	(0.7–2.2)	0.400
Combination of online & offline	**3.8**	**(1.7–8.6)**	**0.001**	1.1	(0.6–2.2)	0.728
Preferred not to answer	0.4	(0.1–1.5)	0.195	0.7	(0.3–1.5)	0.387
Online platform utilization						
General social media only	Ref			Ref		
General social media & gay-specific dating apps	**2.6**	**(1.9–3.6)**	**0.001**	1.0	(0.7–1.3)	0.925
Low social support						
No	Ref			Ref		
Yes	1.4	(0.9–2.2)	0.125	**1.6**	**(1.1–2.5)**	**0.023**
Sex with multiple men at last sex						
No	Ref			Ref		
Yes	**10.9**	**(4.7–25.4)**	**0.001**	**6.1**	**(2.5–14.5)**	**0.001**
Ever engaged in selling or buying sex						
No	Ref			Ref		
Yes	**1.6**	**(1.2–2.2)**	**0.003**	0.8	(0.6–1.2)	0.441
Fully or moderate disclosure of sexuality						
No	Ref			Ref		
Yes	0.8	(0.6–1.1)	0.136	**1.4**	**(1.0–1.9)**	**0.033**
Domain 3: community-level factors						
Internalized homonegativity index (continuous var.)	1.0	(0.9–1.1)	0.911	**1.2**	**(1.1–1.4)**	**0.001**
Ever experienced homosexual-related assaults (lifetime)						
No	Ref			Ref		
Yes	**1.4**	**(1.1–1.9)**	**0.013**	**1.3**	**(1.1–1.9)**	**0.030**
Received HIV/STI health promotion information for MSM in the past 6 months						
No	Ref			Ref		
Yes	0.9	(0.6–1.2)	0.495	**0.7**	**(0.5–0.9)**	**0.020**
Attended HIV-related services in the past 6 months						
No	Ref			Ref		
Yes	1.1	(0.7–1.5)	0.749	1.1	(0.7–1.6)	0.657
Attended STI-related services in the past 6 months						
No	Ref			Ref		
Yes	0.9	(0.7–1.3)	0.679	**0.6**	**(0.4–0.8)**	**0.001**

Bold values indicate statistically significant at P < 0.05

*RR* risk ratio; *STI* sexually transmissible infection

*Including full-time, part-time, and self-employed

## Discussion

To our knowledge, this is the first study in Indonesia to consider multiple levels of socioecological risk factors and assess their associations with individual-level risk of HIV, providing new insights to inform targeted HIV prevention and other support strategies beyond those based on individual-level risk alone. We identified three distinct subgroups of MSM based on shared individual HIV risk behaviors, and showed how these subgroups differed with respect to social and sexual network-level and community-level factors that further influenced risk.

Our study findings indicate that MSM in Indonesia are a heterogenous population with varied patterns of inherent and overlapping risks for HIV transmission. We discovered that although some HIV risks are present in all subgroups, their exposure to multiple and intersecting risks varies. Consistent with previous research, we found MSM who were classified as *Sexual Explorative*, for example, were more likely to engage in SDU and in CAI with casual sex partners [34]. Intersecting risk for other groups were less direct. MSM in the *Navigating Complexities* group were mostly living with HIV, and more likely to have poor mental health and experience a range of socioecological risks likely to drive both mental health and service access outcomes. Mental health issues disproportionately affect MSM due to minority stress [35] and can increase the risk of HIV transmission by diminishing medication adherence [36]. Whilst the *Sexually Moderate* group mostly reported regular partner(s) and sex with only one person, meaningful levels of HIV transmission risk occur in the context of sex between regular partners [37], facilitated in part by the greater likelihood of consistent condom use, which was also reported in our *Sexually Moderate* group.

Our findings provide a richer picture of HIV risk that can inform more holistic approaches to HIV prevention in Indonesia than previous studies describing risk solely based on individual-level risk behavior [38, 39]. For example, MSM identified within the *Sexual Explorative* group had more active roles in their social and sexual networks. Previous research has also shown that the size and density of both social and sexual networks are predictors of HIV risk [30]. Conversely, MSM in the *Navigating Complexity* group reported relatively passive roles in social and sexual networks but exhibited additional demographic and community-level risk. Social and economic disadvantage experienced by the *Navigating Complexity* group—including low social support, being less open about sexuality, internalized homonegativity, experience of homosexual-related assaults, and less exposure to HIV/STI health promotion and STI services—can reduce engagement with HIV care services [40, 41]. With these socioecological factors more prominent, the *Navigating Complexities* group may derive particular benefit from person-centered HIV prevention and care programs in Indonesia, including the integration of HIV and STI testing and treatment services with broader psychosocial support programs.

Our findings should be interpreted in light of several limitations. First, while 75% of the Indonesian general population has access to the internet [42], online surveys limit generalization of findings to MSM without internet access and can be particularly affected by non-response bias. To account for these limitations, we inflated the sample size by 20% beyond that estimated in an a priori calculation to improve the precision and representativeness of survey estimates. Second, our use of HIV service and community-based organizations to recruit participants for this study means the sample represents a subset of MSM who engage in HIV health care, and therefore results may not be generalizable to all MSM in Indonesia. The relatively high service-engaged nature of the sample is evident in the high proportion of self-reported HIV-seropositive status. However, the assignment of HIV-positive status to a specific group in the LCA mitigates potential bias or misinterpretation of our study results. Third, the Indonesian PrEP program was only piloted in 2021, [19] and the Chemsex-INA study did not ask questions about PrEP. Prospective access to PrEP may limit the future validity of findings. Fourth, self-reports may have been influenced by participant recall and social desirability bias. However, the self-administered and online nature of the survey potentially reduced reluctance to answer sensitive questions. Fifth, while there was a possibility of measurement error when posterior probability of latent class membership was used as a predictor in multinomial regression analysis, the study’s high LCA entropy reduced this uncertainty [43]. Finally, due to the cross-sectional study design, our study was unable to establish causality between HIV risk patterns and socio-ecological risk factors.

## Conclusions

The results of this study provide a new and clearer understanding of different HIV risk profiles among MSM and their co-occurrence with specific socioecological factors, which can be used to develop more holistic prevention and care strategies. Differentiated HIV prevention and care approaches are needed to support a diverse MSM population in Indonesia. HIV programs that have focused on individual-level risks should take account of the additional and diverse socioecological conditions that can moderate risk. In addition, HIV-related health promotion should be designed with consideration of vulnerability driven by socioecological factors to enhance their effectiveness in reducing HIV-related risks and support broader psychosocial wellbeing outcomes for MSM in Indonesia. For instance, online outreach may work well to reach MSM within the *Sexual Explorative* group, peer navigation may be more successful at reaching MSM within the *Navigating Complexities* group, and partner notification may be a useful tool to reach MSM within the *Sexually Moderate* group. Finally, the use of LCA adds to the existing literature on the utility of a more nuanced analytical approach to understand HIV risk profiles among MSM in Asian countries in order to improve the effectiveness of HIV interventions [44–47].

### Supplementary Information

Below is the link to the electronic supplementary material.

Supplementary file1 (PDF 102 KB)

## Data Availability

The data that support the findings can be requested from the corresponding author upon reasonable request.
