# Supplementary material for: Characterizing Socioecological Markers of Differentiated HIV Risk Among Men Who Have Sex with Men in Indonesia
Source: AIDS Behav. 2024 Jan 25;28(2):657–68. doi: 10.1007/s10461-023-04253-3 (PMC10876766; doi:10.1007/s10461-023-04253-3)
Supplement: Supplementary file 1 — Supplementary file1 (PDF 102 KB) [file 10461_2023_4253_MOESM1_ESM.pdf]

Appendices – Journal of AIDS and Behavior

Characterizing socioecological markers of differentiated HIV risk among men who have sex with men in Indonesia

Laura Nevendorff[1,2,3§], Alisa Pedrana[1,2], Adam Bourne[4,5], Michael Traeger[1], Eric Sindunata[3], Wawa A. Reswana[6], Rosidin M. Alharbi[6], Mark Stoové[1,2]

[1] Disease Elimination Program, Burnet Institute, Melbourne, Australia

[2] School of Public Health and Preventive Medicine, Monash University, Melbourne, Australia

[3] HIV AIDS Research Center Atma Jaya Catholic University Jakarta, Indonesia

[4] Australian Research Centre in Sex, Health and Society, La Trobe University, Melbourne, Australia

[5] Kirby Institute, University of New South Wales, Sydney, Australia

[6] *Jaringan Indonesia Positive* (The Positive Indonesia Network), Jakarta, Indonesia

[§]Corresponding author: Laura Nevendorff
Burnet Institute, 85 Commercial Rd, Melbourne Australia 3004.
laura.milette@gmail.com, +61414292406

Table 1. Goodness-of-fit indicators comparing class membership models of HIV-related risk factors (N=1413)

Number of Classes	chi^2	p > chi^2	df	AIC	BIC	Entropy
2	1087.6	0.001	25	15333	15463	0.722
**3***	**934.5**	**0.480**	**38**	**15206**	**15403**	**0.816**
4	845.9	0.961	51	15144	15408	0.714
5	783.6	0.999	64	15107	15439	0.655

df= Degree of Freedom; AIC= Akaike Information Criteria; BIC= Bayesian Information Criteria

*Selected based on goodness-of-fit evaluation assessment
